# Nuclear Actin and Lamins in Viral Infections

**DOI:** 10.3390/v4030325

**Published:** 2012-02-28

**Authors:** Jakub Cibulka, Martin Fraiberk, Jitka Forstova

**Affiliations:** Charles University in Prague, Faculty of Science, Department of Genetics and Microbiology, Vinicna 5, 12844, Prague 2, Czech Republic; Email: jakubcib@gmail.com (J.C.); martin.fraiberk@natur.cuni.cz (M.F.)

**Keywords:** viruses, nuclear actin, nuclear lamina, lamin, cytoskeleton, nucleus

## Abstract

Lamins are the best characterized cytoskeletal components of the cell nucleus that help to maintain the nuclear shape and participate in diverse nuclear processes including replication or transcription. Nuclear actin is now widely accepted to be another cytoskeletal protein present in the nucleus that fulfills important functions in the gene expression. Some viruses replicating in the nucleus evolved the ability to interact with and probably utilize nuclear actin for their replication, e.g., for the assembly and transport of capsids or mRNA export. On the other hand, lamins play a role in the propagation of other viruses since nuclear lamina may represent a barrier for virions entering or escaping the nucleus. This review will summarize the current knowledge about the roles of nuclear actin and lamins in viral infections.

## 1. Introduction

Viruses are intracellular pathogens known to employ various host-cell mechanisms to facilitate their replication. The cell cytoskeleton is not an exception. To date, many interactions between viruses and cytoskeleton have been described including virus entry, transport of viral particles in the cytoplasm, and release of progeny virions [1]. 

It would not be surprising if similar interactions between viruses and cytoskeletal proteins also occurred in the nucleus. In fact, there is growing evidence of interactions between certain viruses and two cytoskeletal constituents of the nucleus–nuclear actin and nuclear lamins. After brief characterization of nuclear actin and lamins, respectively, we discuss their role in the replication of viruses from individual families.

## 2. Viruses and Nuclear Actin

For a long time, the existence and function of actin in the nucleus had been rather controversial. Although seen as early as in the 1970s in amphibian oocytes [2] and even described as necessary for transcription on salamander lampbrush chromosomes [3], nuclear actin was long considered to be cytoplasmic contamination or experimental artifact. In the last decade, numerous reports not only confirmed the actin presence in the nucleus, but also showed actin involvement in several crucial nuclear processes. Nuclear actin plays an important role in transcription, transcription regulation, and chromatin remodeling [4,5,6]. Actin is required for the function of all three RNA polymerases [7,8,9]. Moreover, it was shown to bind some pre-mRNA-binding proteins on the nascent transcripts [10,11,12], and even recruit histone acetyl transferases to actively transcribed areas [13]. Nuclear actin is also believed to participate in chromatin remodeling complexes, e.g., SWI/SNF-like BAF complex [14].

Interestingly, myosin isoform I is also located in the nucleus [15]. Nuclear myosin I is essential for RNA polymerase I [9] and RNA polymerase II transcription [16,17], and interacts with the chromatin remodeling complex, WSTF-SNF2h, which participates in rRNA gene transcription [18]. Other studies confirmed the roles of nuclear actin and myosin in the RNA polymerase I transcription, and even suggested mutual cooperation of these two proteins [19,20,21]. 

Despite the evidence of nuclear actin function, its form remains enigmatic. In normal physiological conditions, actin cannot be detected in the nucleus in its polymeric form (e.g., by phalloidin staining) [22,23]. Actin could be present in the nucleus in its monomeric state, but it may also form some non-traditional oligomeric or polymeric conformations. Indeed, there is indirect evidence that nuclear actin can exist in conformations distinct from the cytoplasmic actin [24,25,26]. Nuclear actin may also be involved in nucleoskeletal structures, where it could participate in nuclear transport or maintenance of nuclear shape [27,28,29,30]. Besides actin, many actin-binding proteins (ABPs) and actin-related proteins (ARPs) are also found in the nucleus, where they take part in nuclear processes [30,31]. Finally, it should be noted that nuclear accumulation of actin is also connected with cellular stresses such as heat shock or DMSO treatment [32]. Viral infection naturally also represents a stressful situation, and this should be remembered when discussing subsequent findings regarding viruses and nuclear actin. 

### 2.1. Herpesviruses

Herpesviruses are large enveloped double-stranded DNA viruses with complex structure. The linear dsDNA genomes are located in capsids with icosahedral symmetry. The entire virions are surrounded by a host-cell derived membrane envelope with a number of viral glycoproteins. The capsid and the envelope are divided by an asymmetrical amorphous proteinaceous layer called tegument [33,34].

Herpesviruses replicate in the cell nucleus. Viral replication, late transcription, and formation of viral capsids take place in the ‘replication compartments’ (RCs) [35,36,37,38,39,40], intranuclear structures originally defined by the presence of viral single-strand DNA-binding protein ICP8, and other viral and host factors [39,41,42]. RCs are formed by fusion of smaller pre-replication sites [43] followed by dramatic changes in the nuclear morphology, host chromatin marginalization, and finally nuclear lamina disruption apparently required for virion egress [44,45,46,47]. 

#### 2.1.1. Nuclear Actin in the Assembly and Transport of Viral Capsids

Three members of the *Alphaherpesvirinae* subfamily–herpes simplex virus 1 (HSV-1), herpes simplex virus 2 (HSV-2), and pseudorabies virus (PRV) were shown to induce filament formation in the nuclei of infected cells [48,49]. In the case of PRV infection, the observed filaments had an average length of 3 µm and a diameter of 25–100 nm (rather suggesting bundles of filaments), and associated with the viral capsids, as shown by serial-section block-face scanning electron microscopy (SBFSEM) [48]. Further examination revealed that these filaments consisted of F-actin and their polarity corresponded with the overall polarity of the cell–they formed predominantly on the side of the nucleus facing the Golgi apparatus [48]. The nuclear actin filaments (stained with fluorescent phalloidin) colocalized with the GFP-labeled main capsid protein, VP26, and were required for the formation of GFP-VP26 foci (where the capsids are likely assembled) [48]. The findings of another report suggest that the motion of HSV-1 capsids in the nucleus is active and dependent on actin and myosin [50]. Indeed, nuclear myosin Va strongly colocalized with GFP-labeled capsids of PRV, mainly in the GFP-VP26 foci [48]. Taken together, we can assume that nuclear actin filaments (possibly together with nuclear myosin Va) play a role in the assembly and/or transport of alphaherpesviral capsids.

#### 2.1.2. Nuclear Actin and Morphological Changes of Infected Nuclei

The HSV-1 infection induces dramatic changes in the structure of infected nuclei. Along with the appearance of virus RCs, their growth and fusion, we can also observe marginalization and dispersion of host chromatin and substantial enlargement of infected nuclei (to twice the volume of uninfected nuclei) [44]. Finally, the nuclear lamina is disrupted, and virions escape the nucleus (see later).

In HSV-1-infected cells treated with latrunculin A, reductions in enlargement of nuclei, host chromatin dispersion, and RC maturation were observed in comparison with the untreated control cells. This suggests participation of nuclear actin in the maturation of RCs and accompanying processes. Interestingly, treatment of HSV-1-infected cells with cytochalasin D did not exhibit such reductions (discussed in next subsection) [46]. Besides that, HSV-1 is also able to disrupt the nucleoskeletal structure visualized by GFP-Cdc14B fusion protein [46]. Cdc14B is a phosphatase acting in the cell cycle regulations, which can localize to intranuclear filaments connecting nucleoli and nuclear periphery (and often ending in the vicinity of nuclear pores) [51]. These filaments are about 7 nm in diameter, and their formation is actin dependent [51]. In the course of HSV-1 infection, the filaments are disrupted, and GFP-Cdc14B forms point aggregates in the nuclei [46].

Finally, the sequestration of actin monomers by latrunculin A does not prevent the HSV-1-induced nuclear lamina disruption [46], and this process is therefore probably actin independent.

#### 2.1.3. Monomeric *versus* Polymeric Nuclear Actin

While it seems that the polymeric F-actin plays a certain role in the capsid assembly, it is not clear what form of nuclear actin is responsible for other mentioned phenomena. The morphological changes of HSV-1-infected nuclei are abolished in the presence of latrunculin A but not cytochalasin D [46]. Similarly, latrunculin A but not cytochalasin D was shown to decrease the mobility of HSV-1 capsids in the nucleus [50]. Considering that latrunculin A binds actin monomers (G-actin) and thus inhibits its potential functions, we can assume that the above-mentioned phenomena are G-actin dependent. The disassembly of F-actin by cytochalasin D (binding specifically to the growing end of actin filament) had little effect on the nuclear morphology and capsid mobility [46,50]. Unfortunately, data concerning the effects of actin inhibitors on alphaherpesvirus infectivity are rather inconsistent. Cytochalasin D was shown to reduce PRV infectivity [52], but no reduction in replication after latrunculin A or cytochalasin D treatment was observed for HSV-1 (cytochalasin D in fact markedly increased the infectious titer of HSV-1) [46]. We should also consider the possibility that nuclear actin is distinct in its conformation from the cytoplasmic F-actin, and therefore the effects of these inhibitors may differ. Whatever the functions of nuclear actin, they are rather auxiliary than essential for virus replication, since treatment with actin polymerization inhibitors does not affect replication of HSV-1 [46].

#### 2.1.4. Actin as Part of Herpesviral Virions

Actin can be incorporated into virions of PRV [52,53], human cytomegalovirus (HCMV) [54], murine cytomegalovirus (MCMV) [55], and Kaposi’s sarcoma-associated herpesvirus (KSHV) [56,57]. It is localized predominantly in the tegument [53]. In the case of PRV, it was shown that actin can partly replace the main tegument protein, VP22 [53]. Furthermore, filaments similar to F-actin were observed both in the perinuclear [58] and extracellular virions [34] of HSV-1. These filaments appeared to connect the nucleocapsid with the membrane envelope of the virion [34,58]. Despite many evidences of actin presence in the virions of herpesviruses, its function remains unknown.

### 2.2. Baculoviruses

Baculoviruses are dsDNA viruses infecting invertebrates, mainly insects from orders *Lepidoptera*, *Hymenoptera*, and *Diptera*. Baculoviruses are unique in producing two morphologically distinct infectious viral particles. Budded virions (BV), formed by budding of the nucleocapsid from the host cell cytoplasmic membrane, are responsible for viral spreading between the cells of one individual and inducing systemic infection. Occlusion-derived virus (ODV) gains its envelope in the nucleus of infected cells (most likely from the nuclear membrane invaginations). The virions of ODV are further incorporated into huge paracrystalline proteinaceous matrix formed by protein polyhedrin (nucleopolyhedroviruses) or granulin (granuloviruses). ODV can infect other individuals and is capable of long persistence outside the host cell.

The polyhedral-shaped ODV particle of nucleopolyhedroviruses contains a number of enveloped virions connected by polyhedrin. According to the number of nucleocapsids in one envelope, the nucleopolyhedroviruses are further divided to single nucleopolyhedroviruses (SNPV; one capsid per envelope) and multiple nucleopolyhedroviruses (MNPV; more capsids per one envelope). The granuloviral ODV particle contains only one virion in the ovicylindrical granulin occlusion (baculovirus biology briefly reviewed at [59]).

Replication, transcription, and morphogenesis of baculoviral capsids take place in the cell nucleus. Viral DNA replication occurs in specific intranuclear domains that grow gradually to form the ‘virogenic stroma’. Virogenic stroma occupies most of the nucleus and marginalizes host chromatin [60].

#### 2.2.1. Nuclear Actin Filaments and Nucleocapsid Morphogenesis

More than twenty years ago, cytochalasin D was found to inhibit *Autographa californica* multiple nucleopolyhedrovirus (AcMNPV) replication by preventing proper nucleocapsid assembly in the infected nuclei [61,62,63]. These results suggested that F-actin is somehow involved in this nuclear process, as it was in fact later confirmed [64,65,66].

Nuclear actin filaments form in the AcMNPV-infected cells in the late phase of infection (starting 12 hpi) and are located mainly in the area bordering the virogenic stroma, where they colocalize with the main capsid protein, p39 [64,65]. The experiments with mutated actin resistant to cytochalasin D definitely confirmed that inhibition of proper nucleocapsid morphogenesis induced by this inhibitor was caused by nuclear F-actin disassembly [66].

Other nucleopolyhedroviruses–*Spodoptera frugiperda* MNPV, *Bombyx mori* NPV, *Orgyia pseudotsugata* MNPV, *Lymantria dispar* MNPV, *Anticarsia gemmatalis* MNPV, and *Helicoverpa zea* SNPV–are not able to create infective progeny in the presence of either cytochalasin D or latrunculin A [67]. These findings suggest a conserved mechanism of nuclear F-actin employment in the nucleocapsid morphogenesis of nucleopolyhedroviruses.

#### 2.2.2. Actin Relocalization to the Nucleus

The prerequisite for nuclear actin polymerization in late infection is previous accumulation of sufficient amounts of actin monomers in the nucleus. As described for AcMNPV, this happens already in the early phase of infection with participation of products of six viral genes: ie-1, pe38, he65, Ac004, Ac102, and Ac152 [68]. IE1 and PE38 proteins are immediate-early transcription activators, he65 encodes delayed-early protein, and the products of the remaining genes have not been characterized yet. The product of Ac152 is most likely transactivator of Ac102 and he65 genes. The expression of these six genes is sufficient for G-actin nuclear localization but not for its polymerization [68].

#### 2.2.3. Mechanism of Nuclear Actin Polymerization

The AcMNPV nucleocapsids are able to induce actin polymerization *in vitro* [69] and *in vivo* in the early infection after their release from endosomes [70]. Two actin-binding capsid proteins of AcMNPV were identified: p39 and p78/83 [69].

Study of actin polymerization kinetics using fluorescence recovery after photobleaching (FRAP) revealed the dynamic nature of nuclear F-actin in the AcMNPV-infected cells. Jasplakinolide (stabilizing actin filaments and inhibiting further polymerization) prevented fluorescence recovery in FRAP experiments and lowered the viral infectivity substantially [71]. It means that not only F-actin formation but also its dynamic polymerization plays a key role in the AcMNPV life cycle. The Arp2/3 complex (common host cell actin nucleator) is responsible for nuclear actin nucleation in this step of infection. Arp2/3 is recruited to the nucleus and activated by viral capsid protein p78/83 [71]. Protein p78/83 of nucleopolyhedroviruses contains several highly conserved sequences typical for the Wiskott-Aldrich syndrome protein (WASP) family: proline-rich region, G-actin binding WH2 (WASP-homology 2) domain, and Arp2/3 binding CA (connector and acidic) region [72]. The purpose of WASP proteins and related activators of actin nucleation is activation of Arp2/3 and subsequent actin filament nucleation. Protein p78/83 is therefore probably able to mimic the action of cellular proteins from the WASP family [71]. 

Another viral capsid protein, C42, is essential for nuclear F-actin formation and proper nucleocapsid assembly. This protein mediates translocation of p78/83 into the nucleus using its nuclear localization signal [73]. Moreover, it participates directly in the nuclear actin polymerization, probably via its pocket protein binding sequence (PPBS) [74]. The absence of C42 prevents the nuclear actin filament assembly and correct nucleocapsid morphogenesis even when the nuclear localization of p78/83 is provided artificially [74]. Mutation of the PPBS of C42 does not impair the nuclear translocation of p78/83, Arp2/3, or G-actin, but it blocks the nuclear actin polymerization and reduces viral infectivity [74].

The above-mentioned findings apply to AcMNPV, but the same mechanism of nuclear actin polymerization was also described for *Helicoverpa armigera* MNPV [75]. Taken together with the high level of similarity in WASP-related sequences among different nucleopolyhedroviruses [72], we can assume a general mechanism of nuclear actin utilization valid for all nucleopolyhedroviruses.

#### 2.2.4. F-Actin and the Nuclear Egress of AcMNPV

Recently, one more protein of AcMNPV, VP80, was found to interact with the host nuclear actin [76]. VP80 associates both with nucleocapsids and nuclear actin filaments that connect virogenic stroma and nuclear periphery [76]. VP80 is also indispensable for nuclear export of AcMNPV capsids [77]. Interestingly, this export seems to be actin and myosin dependent. Supported by the fact that VP80 shares sequence homologies with the paramyosin protein family, this may point to nuclear export of nucleocapsids using the actin-myosin complex [76].

### 2.3. Retroviruses

Besides herpesviruses and baculoviruses, two retroviruses have been shown to use nuclear actin in their life cycle. The first is human immunodeficiency virus type 1 (HIV-1) from the genus lentivirus, the second is Mason-Pzifer monkey virus (MPMV) from the genus betaretrovirus. Both viruses need nuclear actin to transport their unspliced mRNAs from the nucleus to the cytoplasm [28,29].

HIV-1 Rev is a viral protein responsible for transport of unspliced and partially spliced viral mRNAs from the nucleus to the cytoplasm. In its amino-terminal region, Rev contains a sequence that serves as nuclear localization signal and also specifically recognizes the hairpin structure present on unspliced viral mRNAs called Rev response element (RRE). The carboxy-terminus of Rev includes the nuclear export signal that interacts with exportin 1 facilitating (together with Ran GTPase) translocation of Rev with bound mRNA to the cytoplasm through the nuclear pore complex. Another factor essential for Rev-dependent export of HIV-1 mRNAs to the cytoplasm of *Xenopus laevis* oocytes is translation initiation factor eIF-5A. eIF-5A interacts with exportin 1, Rev, various nucleoporins, and even actin [28]. Mason-Pfizer monkey virus does not encode any protein similar to Rev, and transport of unspliced mRNAs depends on the host cell factors recognizing specific RNA structure, constitutive transport element (CTE) (mRNA export of HIV-1 and MPMV reviewed at [78]). Export of mRNA using CTE is not dependent on eIF-5A [28].

Nuclear actin filament bundles were observed in the nuclei of transfected HeLa cells that expressed HIV-1 RNAs. These bundles, intersecting the nucleus and pointing to the nuclear envelope, colocalized with gag mRNA, Rev protein, exportin 1, and GTPase Ran. Disassembly of the actin bundles with latrunculin B inhibited nuclear export of gag mRNA, but not of completely spliced tat/rev mRNA or cellular mRNA for glyceraldehyde 3-phospahate dehydrogenase [29]. The necessity of nuclear actin for Rev-dependent (HIV-1) as well as Rev-independent (MPMV) transport of unspliced retroviral mRNAs was further confirmed in microinjection experiments performed with *X. laevis* oocytes and Vero cells [28]. Similar results were surprisingly obtained for the host protein kinase inhibitor (PKI) possessing its own nuclear export signal [28]. The nuclear actin involved in this nuclear export could be in its polymeric, filamentous state [29], but experiments with actin inhibitors point to actin monomers or short oligomers rather than to F-actin [28]. This is also supported by labeling with 2G2 antibody [28] that recognizes the actin conformation specific for the nucleus [24]. Whatever is the case, the actual function and form of nuclear actin in retroviral infections will have to be clarified by further research.

## 3. Viruses and Nuclear Lamins

Lamins are the best known cytoskeletal constituents of the nucleus. They belong to the intermediate filament protein family (class V) and possess the typical structure of intermediate filaments. Mammalian cells produce four main types of lamins: lamin A and lamin C (called A-type lamins) are different splicing products of the same gene, lamin B1 and lamin B2 (B-type lamins) are encoded by two distinct genes. A-type and B-type lamins differ in several characteristics, e.g., isoelectric point and behavior during mitosis. Lamins represent the main components of the nuclear lamina, proteinaceous filamentous layer that is located between chromatin and inner nuclear membrane and contributes significantly to the structural integrity of the nuclear envelope. Nuclear lamina was traditionally described as a regular network of lamin filaments [79], but recent reports revealed more complicated structure dependent on the lamina composition and cell type [80]. Besides the nuclear lamina, lamins were also found as spots in the nucleus interior [81,82,83] and in the case of lamin A even as a part of intranuclear filaments [84,85,86,87]. Today, lamins are recognized to play many roles in different nuclear processes including replication, transcription, chromatin organization, and others [88]. Mutations in genes for nuclear lamina components (mainly lamin A gene) are associated with a variety of human diseases called laminopathies [89].

### 3.1. Herpesviruses

Thanks to the size of nucleocapsids of herpesviruses (~125 nm in diameter for HSV-1), the nuclear envelope represents a significant barrier on their way out of the nucleus. Only particles up to 39 nm in diameter can be transported through the nuclear pore [90], and the size of nuclear lamina fenestrations is approximately 15 nm [80]. Thus, either enlargement of nuclear pores or changes in the lamina structure are necessary for successful release of herpesvirus nucleocapsids to the cytoplasm. According to the now widely accepted ‘envelopment-deenvelopment-reenvelopment’ hypothesis, the nucleocapsids bud through the inner nuclear membrane to perinuclear space. This primary envelope subsequently fuses with the outer nuclear membrane, and naked nucleocapsids escape to the cytoplasm, where they gain a new envelope derived from the endoplasmic reticulum (ER) or Golgi apparatus membranes [91,92]. This model assumes that herpesviruses are able to induce restructuring or disassembly of nuclear lamina that would normally prevent the direct contact of nucleocapsids with the inner nuclear membrane.

Many studies confirmed the involvement of herpesviruses in the changes of nuclear lamina structure. HSV-1, HSV-2, MCMV, HCMV, and Epstein-Barr virus (EBV) use similar mechanisms to disrupt the nuclear lamina and release the virions from the nucleus [93]. These mechanisms utilize viral as well as host factors and will be discussed in detail on the example of HSV-1, and briefly described for other herpesviruses.

#### 3.1.1. HSV-1

Cells infected with HSV-1 exhibit different fluorescent profiles of nuclear lamins and lamin B receptor, suggesting thinning and partial disassembly of the nuclear lamina [45]. These findings were obtained by observing living cells producing proteins lamin B receptor, lamin A, and lamin B2 fused with GFP and also by indirect immunofluorescence using antibodies against lamin A/C, lamin B1, and lamin B2. Furthermore, during infection, the rate of lamin B receptor diffusion in the inner nuclear membrane and solubility of lamin A are significantly increased [45]. HSV-1 infection also induces an overall decrease in the amount of lamins in infected cells [45]. 

**Figure 1:**
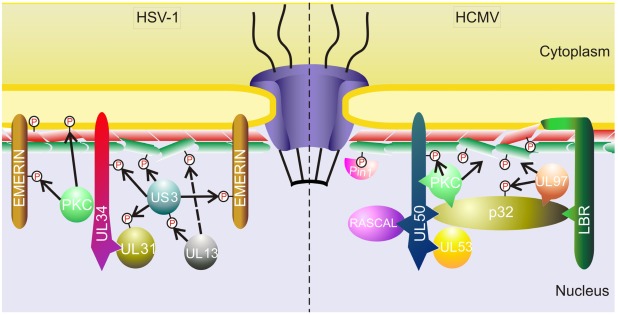
Nuclear egress complex of herpes simplex virus 1 (HSV-1) and human cytomegalovirus (HCMV). The lamin proteins are depicted as two layers beneath the nuclear membrane (lamin B in red, lamin A/C in green). The red “P” in circle marks phosphorylated proteins, actions of individual protein kinases are represented by black arrows. The dashed arrow suggests hypothetical phosphorylation of lamins by pUL13 kinase (proved for HSV-2). The lamin B receptor protein is abbreviated as “LBR”. Besides PKC, other cellular kinases may be involved in phosphorylation of nuclear lamina components.

Disruption of nuclear lamina by HSV-1 is coupled with RC maturation [47] and involves action of both viral and host proteins. These include viral proteins associated with the nuclear envelope together with viral as well as cellular kinases. All these proteins form the ‘nuclear egress complex’ (Figure 1, left) and work in concert to facilitate nuclear lamina breach and thus the egress of new virions from the nucleus.

The viral transmembrane protein necessary for primary envelopment, pUL34 [94], and the viral phosphoprotein found to be associated with the nuclear matrix, pUL31 [95], form together a complex that associates with the inner nuclear membrane [96,97,98]. This localization of pUL34 and pUL31 is observable only when expressed together (infection with viruses that had deleted either gene led to mislocalization of the remaining protein) [96,97]. Coexpression of UL34 and UL31 genes alone is sufficient for pUL34 and pUL31 localization to the nuclear envelope [96]. However, for even distribution of the complex along the nuclear rim, viral kinase pUS3 is required. When pUS3 is absent, pUL34 and pUL31 are unevenly distributed throughout the nuclear rim and associate with the nuclear membrane invaginations containing clusters of primarily enveloped virions [96,97]. The correct localization of pUL34 also depends on the presence of the lamin A/C itself [99].

Besides the role in primary envelopment of virions [94,96,97], the pUL34-pUL31 complex is responsible for nuclear lamina disruption. Viruses lacking UL34 or UL31 genes are not capable to induce changes in the immunoreactivity of lamin A/C and lamin-associated polypeptide 2, typical of cells infected with the wild-type virus [47]. Additionally, the UL34 gene is also required for disruption of lamin B [100]. The role of pUL31 and pUL34 in the nuclear lamina rearrangement is further supported by findings that both bind lamin A/C directly and that overexpression of either of them leads to partial lamin A/C relocalization [101].

Whether HSV-1 induces only conformational changes or even disruption of nuclear lamina was examined by different antibodies directed against lamin A/C. Staining with monoclonal antibody against the tail domain of lamin A/C exhibited significant reduction in the infected cells dependent on the pUL34 and pUL31 presence. Polyclonal antibody recognizing epitopes in the rod domain of lamin A/C showed decreased lamin staining even in the absence of pUL31 (but not pUL34). Surprisingly, labeling with the third polyclonal antibody did not exhibit any differences between infected and uninfected cells. Based on these experiments the authors suggested that the alterations in lamin staining during HSV-1 infection were caused by conformational changes in the nuclear lamina rather than its direct disintegration [101]. However, observations in cells producing GFP-lamin A fusion protein showed that HSV-1 caused real perforation of nuclear lamina dependent on the presence of pUL34 and pUL31 [46].

Proteins pUL34 and pUL31 are indispensable not only for nuclear lamina disruption, but also for characteristic nuclei enlargement [46]. 

In HSV-1-infected cells, viral serine/threonine kinase, pUS3, colocalizes with pUL34 and pUL31 on the nuclear envelope and also associates with perinuclear virions [97]. pUS3 can phosphorylate pUL34 [102] and pUL31 [103] and its activity is needed for even distribution of the pUL34-pUL31 complex in the inner nuclear membrane [96,97]. If pUS3 is missing or catalytically inactive, primarily enveloped virions are concentrated in the invaginations of perinuclear space into the nucleoplasm. In these areas, pUL34 and pUL31 accumulation and large perforations of nuclear lamina can be detected [97,100,104]. This effect is probably caused by prevention of pUS3-mediated phosphorylation of pUL31 [103]. The damage to nuclear lamina is greater in the absence of pUS3, suggesting that some negative regulation between the action of pUS3 and pUL34-pUL31 complex exists. This would be in agreement with findings that coexpression of pUS3 (catalytically active) and pUL34 leads to less dramatic changes in the nuclear lamina than individual expression of either gene [100]. Deletion of US3 gene decreases viral infectivity, although surprisingly not in all cell types [97,102].

Apart from the indirect role in the nuclear lamina disruption via regulation of pUL34 and pUL31 activity, pUS3 was shown to directly phosphorylate lamin A/C on several sites *in vitro* and *in vivo* [104]. Moreover, pUS3 alone can increase lamin A/C solubility and induce some defects in the nuclear lamina [100,104]. Interestingly, its kinase activity is not required to disrupt lamins in transfected cells [100]. 

HSV-1 also causes structural changes of proteins of the inner nuclear membrane that are associated with nuclear lamina such as lamin B receptor [45] and lamin-associated polypeptide 2 [47]. Another inner nuclear membrane protein affected by HSV-1 infection is emerin. During infection, emerin is delocalized and exhibits increased mobility [105,106]. This is due to its phosphorylation by host protein kinases [105,106], including protein kinase C (PKC) δ [106]. However, the involvement of PKCδ was later disputed [107]. Virus pUS3 kinase participates in emerin phosphorylation too [105,106], although possibly indirectly by modulating cellular kinase(s) activities [105]. Hyperphosphorylation of emerin is also partially dependent on the presence of pUL34, which is able to bind emerin and recruit cellular kinase(s) [106].

Another kinase participating in nuclear lamina disintegration is a product of the UL13 gene, highly conserved herpesviral serine/threonine kinase. pUL13 is capable to phosphorylate the pUS3 kinase and its deletion leads to a similar phenotype as deletion of US3, *i.e.*, changes in the localization of pUL34 and pUL31 in the inner nuclear membrane [108]. Nevertheless, it is not clear whether pUL13 influences pUL34-pUL31 localization directly or via phosphorylation of pUS3 [108]. It is also worth mentioning that pUL13 of HSV-2 directly phosphorylates nuclear lamins and causes their redistribution [109].

In the course of HSV-1 infection, PKC is concentrated in the vicinity of nuclear envelope [110]. This relocalization occurs between 8 and 12 hours post infection and depends on the presence of the pUL34-pUL31 complex in the nuclear envelope [110]. Viral kinase pUS3 is responsible for the even distribution of PKC along the nuclear rim because it influences the distribution of pUL34-pUL31 in the same way [110]. Two isoforms of PKC–PKCα and PKCδ–are relocalized to the nuclear envelope coincidently with increased lamin B phosphorylation [110]. Lamin B could be phosphorylated directly by PKC and/or other cellular or viral kinases [107,110]. The PKC activity is essential for HSV-1 infection since inhibition of all PKC isoforms causes substantial reduction of viral replication, accumulation of virus particles in the nuclei, and overall decrease in the amount of viral capsids in the infected cells. On the other hand, specific inhibition of conventional PKCs (including PKCα) or PKCδ does not inhibit viral replication [107]. This indicates either functional redundancy of these isoforms or involvement of other PKC forms in the viral life cycle.

#### 3.1.2. Other Herpesviruses

Individual members of the *Herpesviridae* family share substantial resemblance concerning the mechanisms of nuclear lamina disruption. Proteins homological to pUL34 and pUL31 were described for HSV-2 [111,112], PRV [113,114], HCMV [115], MCMV [116], and EBV [117,118,119]. These proteins are, like in HSV-1, responsible for primary envelopment and release of virions from the nucleus. The direct role in the changes of nuclear lamina, including PKC relocalization, was proved for pUL50 and pUL53 of HCMV [115,120,121], although, unlike HSV-1 pUL34, pUL50 alone is able to recruit PKC [121] and even can be phosphorylated itself by this kinase [120]. During MCMV infection, proteins M50/p35 and M53/p38 recruit PKC to the nuclear rim for lamin phosphorylation and lamina dissolution [116]. BFLF2 and BFRF1 proteins of EBV were shown to interact with lamin B [119].

Homologs of HSV-1 pUL13 kinase (‘conserved herpesviral kinases’), pUL13 of HSV-2, pUL97 of HCMV, and BGLF4 of EBV, participate in direct phosphorylation of lamins and disruption of the nuclear lamina [109,121,122,123,124,125,126]. Remarkably, pUL97 and BGLF4 apparently imitate the activity of cellular cyclin-dependent kinase 1, which is responsible for the nuclear lamina breakdown during mitosis [123,126]. Furthermore, the pUL97-mediated phosphorylation of lamin A/C at Ser22 creates a binding motif for the cellular peptidyl-prolyl cis/trans-isomerase Pin1 [125]. During HCMV infection, Pin1 is concentrated by the nuclear lamina in a manner dependent on the protein kinase activity [125]. Pin1 could contribute to nuclear lamina reorganization by inducing conformational changes of lamins [125].

Another cellular protein involved in the HCMV-induced nuclear lamina disruption is p32 protein. This protein recruits pUL97 kinase to the lamin B receptor and is itself phosphorylated by pUL97 [122]. Moreover, p32 also directly interacts with pUL50 and PKC [120,121]. In addition, the recently characterized protein of HCMV, RASCAL (nuclear rim-associated cytomegaloviral protein), was also identified to be involved in the nuclear egress complex, likely via its interaction with pUL50 [127]. The nuclear egress complex of HCMV is depicted in figure 1, right.

Alphaherpesviruses have homologs of HSV-1 pUS3 kinase as well, and they share some functional similarities – for example, pUS3 of PRV influences pUL34 localization in the same way as its HSV-1 counterpart [128] and pUS3 of HSV-2 changes the pattern of emerin hyperphosphorylation [105]. Interestingly, HSV-2 pUS3 exhibits marked differences from HSV-1 pUS3 in its catalytic functions, e.g., it does not control the localization of the nuclear egress complex [129].

Even though the ways of interactions of viral proteins with nuclear lamins slightly vary between individual herpesviruses, they all result in nuclear lamina disruption, thus confirming the role of nuclear lamina as a major obstacle to herpesviral replication.

### 3.2. Other Viruses

Although herpesviruses are by far the most extensively studied virus family regarding their interactions with nuclear lamins, studies describing the interplay between lamins and other viruses are slowly emerging. It seems that, similarly to herpesviruses, the nuclear lamina represents a barrier for all these viruses, and hence they evolved mechanisms to overcome it. However, we have to be more careful about making any definite conclusions since there is only one or a few reports concerning lamins for each below-mentioned virus family.

#### 3.2.1. Retroviruses

HIV-1 requires nuclear actin for nuclear export of its unspliced mRNAs, but there are also evidences for HIV-1 interactions with nuclear lamina. Viral protein Vpr induces perforations in the nuclear envelope corresponding to the sites with defects in nuclear lamina [130]. These perforations lead to mixing of cytoplasmic and nucleoplasmic content, including cell cycle regulators. The authors hence deduce that these defects consequently result in the cell cycle arrest in G2 phase, which is a known effect of the Vpr protein [130]. Apart from that, this action of Vpr could facilitate the nuclear entry of HIV preintegration complex [130,131].

#### 3.2.2. Polyomaviruses

Polyomaviruses are small non-enveloped tumorigenic viruses with circular double-stranded DNA genome. The best studied representatives of the *Polyomaviridae* family are simian virus 40 (SV40), mouse polyomavirus (MPyV), and human pathogens JC virus and BK virus. In the last several years, seven additional human polyomaviruses have been discovered, including Merkel cell polyomavirus associated with rare but aggressive cancer of Merkel cells.

Upon cell entry, polyomaviruses are transported through the endosomal pathway to the ER [132,133,134,135,136]. Infection of MPyV is dependent on acidic pH of the endosomes [137]. Qian *et al*. proposed that MPyV is transported first to the endolysosome, and there the polyomavirus ganglioside receptor stimulates sorting of MPyV to the ER [138]. The precise mechanism controlling the transport of MPyV from the plasma membrane to the ER remains to be clarified. Even less clear is the mechanism by which polyomaviruses deliver their genomes into the cell nucleus. Based on electron microscopy analyses, early papers suggested that SV40 [139] and MPyV [140] enter the cell nucleus bypassing nuclear pores by fusion of vesicles carrying virions directly with the nuclear envelope. At present, two models of polyomavirus trafficking from ER into the nucleus are discussed. The first model presumes that partially disassembled virions are translocated (by an as yet unknown mechanism) from ER to the cytosol and enter the nucleus via nuclear pores. Although it has never been proved, several findings support this hypothesis [141,142,143,144,145]. 

Alternatively, a recent report on SV40 suggests a model, in which the genomes are delivered from the ER directly to the nucleus. During cell entry, SV40 induces transient changes in the structure of the nuclear envelope accompanied by fluctuations in the lamin A/C protein level, accumulation of lamin A/C in the cytoplasm, and dephosphorylation of a specific lamin A/C epitope [146]. These changes culminate 6-8 hours post infection, just prior to and during nuclear entry of the viral genome, and seem to be caspase-6 dependent. Interestingly, these alterations in nuclear envelope structure occur exclusively during infection of non-proliferating cells, and the pentamers of the major capsid protein, VP1, are sufficient to induce them [146]. Interaction of VP1 protein with nuclear lamina was also observed during the expression of MPyV VP1 in mouse fibroblast cells (our unpublished results). Along with the lamina, the nuclear membrane represents a natural barrier for polyomavirus infection. *In vitro* studies on the minor structural proteins, VP2 and VP3, of SV40 [147] and of MPyV [148,149] showed that they are able to bind, insert into, perforate and even fuse cell membranes. Thus, in all the above-discussed models, the minor structural proteins might be key actors helping virions pass through ER and/or inner nuclear membrane.

#### 3.2.3. Parvoviruses

The Minute virus of mice (MVM) is a small non-enveloped ssDNA virus that belongs to the *Parvoviridae* family and replicates in the cell nucleus. In the early phase of infection, prior to the nuclear entry, MVM induces transient breaks in the nuclear envelope accompanied by changes in the lamin A/C immunostaining [150]. Moreover, the gaps in lamin staining are coincident with the antibody-labeled virus [150]. Further examination revealed that host caspases are involved in MVM-induced nuclear envelope breakdown. In particular, basally active caspase 3 is relocalized to the nucleus, where it cleaves nuclear lamins (most likely lamin B2) [151]. These findings suggest that parvoviruses, despite their small size (ca 26 nm in diameter), do not import their genome into the nucleus via the nuclear pore, and instead they cause partial breaks in the nuclear envelope to facilitate nuclear entry. Consistently with this unusual model, capsids of adeno-associated virus 2 (AAV2) were shown to enter purified nuclei independently of nuclear pore complexes [152].

## 4. Conclusions

In this review, we wanted to present the current knowledge on the significance of nuclear actin and lamins for various viruses. Despite the increasing number of reports dealing with this topic, many issues remain unresolved. First of all, we still know very little about the form of nuclear actin even in normal, uninfected cells, and that makes our understanding of its employment by viruses more difficult. Secondly, the most findings presented in this review refer to only two virus families–herpesviruses and baculoviruses. Data on other discussed viruses is based on a few studies only. It would be very surprising if these viruses were the only ones interacting with host nuclear actin or lamins. In fact, it is reasonable to expect that other viruses replicating in the nucleus will soon extend this list. To conclude, the participation of both lamins and nuclear actin in the viral life cycle represents a relatively unexplored but very promising area of research that can tell us much about the ability of viruses to deal with the host cell environment. 

## Acknowledgments

The work was generously supported by the grant SVV-2012-265202 (J.C.), by the grant of Ministry of Education, Youth and Sports of the Czech Republic, n. MSM0021620858 (J.F.), and by the Grant Agency of the Czech Republic, n. P304/10/1511 (M.F.). We are grateful to Sarka Takacova for her assistance in the preparation of this manuscript.

## Conflict of Interest

The authors declare no conflict of interest. 
